# Mutational Analysis of cj0183 *Campylobacter jejuni* Promoter

**DOI:** 10.1007/s00284-013-0420-8

**Published:** 2013-07-25

**Authors:** Agnieszka Salamasznska-Guz, Marta Grodzik, Danuta Klimuszko

**Affiliations:** 1 Department of Pre-Clinical Sciences, Warsaw University of Life Sciences, Warsaw, Poland; 2 Department of Animal Nutrition and Feed Science, Warsaw University of Life Sciences, Warsaw, Poland

## Abstract

Gene-nominated *cj0183* was identified in *Campylobacter jejuni* NCTC 11168 and in two human isolates 81116 and 81-176. It encodes a protein which shows partial homology to TlyC of *Brachyspira hyodysenteriae*. The aim of this work was to determine the mechanisms of gene regulation by cloning DNA fragments lying upstream of the *cj0183* gene. The β-galactosidase activity determined for the strain harboring the plasmid with the fragment upstream of *cj0183* indicated the presence of a promoter in this DNA region. Mutations in *cj0183 *-10 region, -16 region, and -35 region resulted in changes in gene transcription.

## Introduction

*Campylobacter jejuni* is a major cause of bacterial diarrhea; yet, in comparison to other enteric pathogens still little is known about gene regulation in this bacteria. Only a few *C. jejuni* promoters have been experimentally confirmed so far [6, 7, 12, 17]. The *C. jejuni* operon structure is not well described. In silico analysis of the *C. jejuni* genome by Petersen et al. [10] revealed that the operon structure may be more expanded than previously described. The A+T content of the *C. jejuni* genome is very high (70 %) and genes from this bacterium are often difficult to clone and analyze in *Escherichia coli* [17]. Lack of expression in *E. coli* is mostly due to the absence of specific factors required for transcription. The consensus of the *C. jejuni* promoter sequence differs from the σ^70^ promoter consensus sequence of *E. coli* and other bacteria. It has been determined that the *C. jejuni* promoter contains three conserved regions: a -10 region (TATAAT) similar to *E. coli*, a -35 region (TTnAAGTnTT) completely different than in *E. coli*, and a -16 region (TTTTTTTG), which is typical for gram-positive bacteria and similar to the promoter found in *E. coli* promoters, named the extended -10 region [5, 9, 14, 17].

According to the suggestion of Wooldridge et al. [16], the product of the *cj0183* gene found in the *C. jejuni* NCTC 11168 genome is implicated in colonization of epithelial cells of the chicken gut. Structure of the encoded protein contains domains characteristic for proteins with hemolytic activity. The *cj0183* gene was found in all sequenced *C. jejuni* strains and all of the 69 *C. jejuni* and 16 *Campylobacter coli* PCR-tested strains isolated from chicken, dog, and pig stool samples [Sałamaszyńska-Guz, data not published]. The *cj0183* gene was cloned to determine the role of the encoded protein in colonization of epithelial cells of the human gut. In vitro studies showed that mutation in the *cj0183* gene does not influence adhesion to the Caco-2 cell line. Moreover, mutation in the *cj0183* gene did not affect the hemolytic activity of the respective strains [11, 16]. Findings of Carvalho et al. [2] indicate that the TlyC protein from *Leptospira* (homolog of Cj0183) may contribute to binding to the extracellular matrix during host infection.

As little is known about the *C. jejuni* gene promoters, we decided to widen the knowledge concerning the function of specific *C. jejuni* promoter regions by analyzing the promoter of the Cj0183-encoding gene. In order to determine the regulation mechanism of this gene its upstream region was cloned in the pMW10 shuttle vector directly before the *lacZ* gene lacking its own promoter. The resulting recombinant plasmid was introduced into the *C. jejuni* 28C strain, in which the expression of the *lacZ* gene was studied. Consensus of the promoter sequence was determined after sequencing the cloned fragment that activated *lacZ* expression. The function of different motifs in the promoter region was also investigated.

## Materials and Methods

### Bacterial Strains, Plasmids, Media, and Growth Conditions

Bacterial strains and plasmids used in this study are listed in Table 1. The *C. jejuni* 28C strain was grown in microaerobic conditions at 42 °C on Mueller–Hinton (MH) agar (bioMérieux) containing 5 % (v/v) sheep blood. Strains were grown on media supplemented with kanamycin (Km; 25 μg/ml). *E. coli* strains were grown at 37 °C in LB broth or on LB agar (BIOCORP) supplemented with Km (25 μg/ml).

**Table 1:** Bacterial strains and plasmids used in this study

Strain or plasmid	Relevant characteristics	Source or reference
Strain		
*C. jejuni* 81-176		Korlath et al. [8]
* C. jejuni* 28C	Isolate from poultry	Department of Microbiology, Warsaw University of Life Sciences
* E. coli* DH5α	F– Φ80lacZΔM15 Δ(lacZYA-argF) U169 recA1 endA1 hsdR17 (rK–, mK+) phoA supE44 λ– thi-1 gyrA96 relA1	Invitrogen
Plasmid		
pMW10	Shuttle vector, replicating in *C. jejuni* and *E. coli*; Km^r^	Wösten et al. [17]
p0183 (WT)	pMW10 vector with cloned 550 bp DNA fragment of the *cj0183* (fragment comprising the 450 bp region upstream of *cj0183*)	This study
pΔ-35	p0183 vector with deletion of the -35 promoter region	This study
pΔ-35-16	p0183 vector with deletion of the -35-16 promoter region	This study
pΔ-35-16-10	p0183 vector with deletion of the -35-16-10 promoter region	This study
pSubs/restr-10	p0183 vector with substitution of the -10 promoter region by GGATCC sequence	This study
pSubs/restr-16	p0183 vector with substitution of the -16 promoter region by AGATCT sequence	This study
pSubs/cons -16	p0183 vector with substitution of the -16 promoter region by *Campylobacter* *jejuni* -16 consensus sequence TTTTTTG	This study
pSubs/cons -35	p0183 vector with substitution of the -35 promoter region by the *Campylobacter* *jejuni* -35 consensus sequence TTTAAGTATT	This study

*Km* ^*r*^ kanamycin resistant

### DNA Manipulations

Plasmid and chromosomal DNA extraction, restriction enzyme digestions, DNA ligation, agarose gel electrophoresis, and transformations were performed by standard procedures using enzymes supplied by Fermentas. Isolation of DNA from agarose gel was performed according to the manufacturer’s instructions (A&A Biotechnology).

Oligonucleotide primers which were synthesized by Genomed S.A. Primer sequences are given in Table 2. DNA sequencing was performed by Genomed S.A.

**Table 2:** Primers used in this study

Primer	Sequence 5′ → 3′
0183P1	GGATCCTGAGGATAATCGCAAAG ^1^
0183P2	AGATCTAGCAAGTGCAACAACAACCA ^2^
M1	TTTTTTCTTGTATTTTATAAGAAAAAACTAAGACTTATAAGAAATATTTTTCTC
M35	AAATTTACTTTTAATTGTGTATAATAAGGGTTAATAATATTTTATTTCTTAAGG
M35-16	GTATAATAAGGGTTAATAATATTTTATTTCTTAAGGAGCTTCATTG
M35-16-10	AAGGGTTAATAATATTTTATTTCTTAAGGAGCTTCATTGG
Mrest-10	GGATCCCACAATTAAAAGTAAATTTAATATTTGAATTTTTTCTTGTATTTTATAAGAAAAAACTAAGACTTATAAGAAATATTTTTCTC ^1^
Mrest-16	AGATCTGTAAATTTAATATTTGAATTTTTTCTTGTATTTTATAAGAAAAAACTAAGAC ^2^
Mcons-16	CCAAAAAAAAGTAAATTTAATATTTGAATTTTTTCTTGTATTTTATAAGAAAAAACTAAGAC
Mcons-35	TTTAAGTATTAAATTTACTTTTAATTGTGTATAATAAGGGTTAATAATATTTTATTTCTTAAGG
183-6FAM	CACACCATAGGCATAGAAT

Underlined letters indicate restrictase recognition sequences introduced for cloning purposes: ^1^ *Bam*HI and ^2^ *Bgl*II

### Reporter Gene Assays

The *C. jejuni* promoter probe vector pMW10 contains a promoterless *lacZ* gene, and was previously used to quantify promoter activity in *C. jejuni* [17]. An overlapping fragment containing the *cj0183* start codon and part of the upstream region was amplified by PCR using specific primers (Table 2) and *C. jejuni* 81-176 genomic DNA as template. The amplified PCR product was cloned into pMW10 using *Bam*HI and *Bgl*II sites. The resulting plasmid was named p0183 (WT). Constructs were transformed into *E. coli* DH5α and *C. jejuni* 28C strains.

### Site-Directed Mutagenesis

Standard-purified mutagenesis primers were designed following the QuickChange™ II XL site-directed mutagenesis kit guidelines (Stratagene). Mutant Δ -35 was constructed using primers M1 and M35; mutant Δ -35-16—primers M1 and M35-16; mutant M35-16-10—primers M1 and M35-16-10; mutant Subs/restr -10—primers M35-16-10 and Mrest-10; mutant Subs/restr -16—primers M35-16-10 and Mrest-16; mutant Subs/cons -16—primers M35-16-10 and Mcons-16; mutant Subs/cons -35—primers M1 and Mcons-35. Primer sequences are presented in Table 2. PCR products of the mutagenesis reactions were digested with *Dpn*I and purified using the DNA Clean-Up kit (A&A Biotechnology). Subsequently, products were phosphorylated, ligated and used for electroporation of *E. coli* DH5α and *C. jejuni* 28C.

### Transformation of *Campylobacterjejuni*

*Campylobacter jejuni* electrocompetent cells prepared as described by Wassenaar et al. [15] were mixed with plasmid DNA (0.5–5 μg). Electroporation was performed in Electro Cell Manipulator ECM 600 by applying 50 μF, 126 Ω, and 1.25 kV. Transformants were grown under microaerobic conditions at 42 °C on MH plates for 5 h and then plated on BHI agar plates supplemented with 5 % sheep blood and a selective antibiotic—Km. Transformants were grown for 2–5 days.

### β-Galactosidase Assay

β-Galactosidase activity in *E. coli* and *C. jejuni* was measured by conversion of *o*-nitrophenyl-β-d-galactopyranoside into nitrophenol as described by Wösten et al. [17]. ß-Galactosidase activities were expressed in Miller units based on three independent experiments.

### Primer Extension

Total RNA of wild-type *C. jejuni* strain 81-176 was isolated using the RNA protect RNeasy Mini kit (Qiagen) followed by DNase treatment. cDNA synthesis was performed using Fermentas RevertAid First Strand cDNA Synthesis Kit according to the manufacturer’s instruction. RT-PCRs were performed using the 183-6FAM primer (Table 2) and products were analyzed using Peak Scanner™ Software v 2.0 Applied Biosystems.

### Sequence Data Analysis

Each construct which was sequenced by Genomed S.A. Amino acid sequences were aligned using Clustal W. Computational search of wild genome promoters was performed using RSAT (http://rsat.uib.ac.be/rsat) [3, 13].

## Results and Discussion

### Determination of β-Galactosidase Activity

The β-galactosidase activity assay was performed in *E. coli* DH5α and *C. jejuni* 28C cells, harboring the pMW10 recombinant plasmid, p0183 (WT), carrying an insert with the probable promoter region of the *C. jejuni* 81-176 *cj0183* gene amplified using 0183P1 and 0183P2 primers. *E. coli* DH5α and *C. jejuni* 28C strains with the pMW10 plasmid alone served as negative controls.

In accordance with earlier reports [17], a significant variation of β-galactosidase activity between *E. coli* and *C. jejuni,* due to differences in their promoter sequences, was observed (*P* < 0.05). Activity of the enzyme was considerably lower in *E. coli* cells harboring the p0183 (WT) recombinant plasmid (39.7 ± 12.1) compared to the activity detected in *C. jejuni* (619.1 ± 93.7). Findings comply with literature data and result from differences in the genome G+C content of these bacteria, which reflects also dissimilarities in the structure of promoter regions [17]. Based on the β-galactosidase activity assay in *E. coli* and *C. jejuni* strains harboring the constructs, it seems that the upstream DNA sequence of the *cj0183* gene most probably contains a promoter sequence.

### Analysis of Nucleotide Sequences Upstream of the *cj0183* Gene

**Fig. 1:**
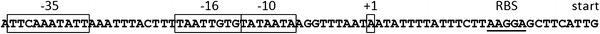
Mapping of the transcriptional start site of *cj0183* gene. Putative location of the *cj0183* transcriptional start point determined by primer extension assay. Putative -35, -16, and -10 sequence motifs of a σ^70^-promoter and start codon of *cj0183* are* boxed*. The ribosomal binding site is* underlined*

The coding and promoter sequences of the *cj0183* gene are highly conserved among sequenced *C. jejuni* genomes (99–100 % for coding and 100 % for promoter sequence). Based on bioinformatic analysis (RSAT) it seems highly probable that *cj0183* remains under the control of the σ^70^-recognized promoter [13]. Sequence of the upstream region of *cj0183* showed no homology to sequences recognized by other sigma factors of *C. jejuni*. The *cj0183* promoter contains three typical conserved promoter motifs: -10, -16, and -35 [17], at an appropriate distance upstream from the transcriptional start site (Fig. 1). Position of the first T in the -10 motif of the predicted promoter in relation to the start codon is 42 bp; majority of -10 motifs predicted by Petersen et al. [10] start 30–43 bp upstream of the start codon.

**Fig. 2:**
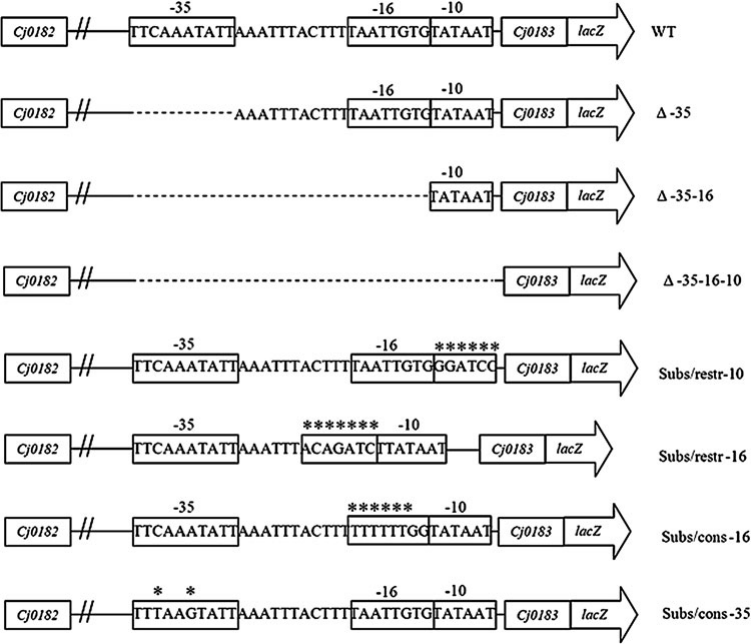
Schematic representation of the mutant constructs. WT represent wild-type promoter. Putative -35, -16, and -10 sequence motifs of a σ^70^-promoter and start codon of *cj0183* are* boxed*.* Asterisks* indicate nucleotide substitutions

**Fig. 3:**
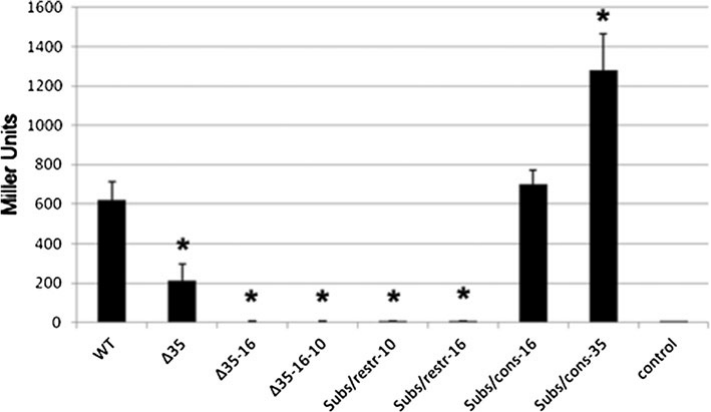
Mutational analysis of the *cj0183* promoter region. Quantitative analysis of *lacZ* expression of the *cj0183* promoter deletion/substitution vector series in *C. jejuni*, including the construct carrying the wild-type *cj0183* promoter (WT) as a positive control and the empty vector (control) as a negative control. Reactions were performed in triplicate, and standard deviations are marked by* error bars*. *P > 0.05

We constructed various deletions mutants within the assumed promoter region by removing: (i) the whole promoter region (Δ-35-16-10), (ii) -16 and -35 motifs (Δ-35-16), or (iii) only the -35 motif (Δ-35) (Fig. 2). All of these deletions significantly affected the activity of the β-galactosidase reporter gene (Fig. 3). Deletion of the -35-16-10 and -35-16 region abolished completely and deletion of the -35 region decreased threefold β-galactosidase activity, indicating that these regions are involved in gene transcription under standard laboratory conditions.

### Genome Analysis of Putative *Campylobacter jejuni* Promoters and Experimental Validation of *cj0183* Promoter Motifs

We used RSAT to perform genome analysis of putative *C. jejuni* 81-176 strain promoters under the following criteria: (i) both strands of DNA were scanned, (ii) TnAAGTnnn and TATAAT were the conserved -35 and -10 motifs, respectively, (iii) only hits located in noncoding sequences were counted, (iv) spacers between the -10 and -35 motifs in *C. jejuni* contained 15–19 bp [3, 13, 17]. We predicted promoter regions upstream of 146 reading frames (from 100 bp upstream to 1 bp downstream of the annotated start codon). Among them, 133 promoters were located on the chromosome, 10 on the Vir plasmid and 3 on the Tet plasmid. Our results indicate that promoters with a 18 bp (*n* = 55) spacer are dominant, which is in contrast to the study of Wösten et al. [17] reporting that promoters with a 17 bp spacer were the most prevailing. In our study, we determined that the promoter of the *cj0183* gene has a 19 bp spacer (Table 3).

**Table 3:** Spacer length of *C. jejuni* promoters

Length of spacer region	Number of promoters					
	According to Wösten et al. [17]			According to this study		
	All promoters	TG promoters	Non-TG promoters	All promoters	TG promoters	Non-TG promoters
All	21	7	14	146	19	127
15	6	3	3	19	2	17
16	3	2	1	31	7	24
17	7	2	5	31	4	27
18	3	–	3	55	5	50
19 (*cj0183*)	1	–	1	10	1	9

The importance of putative *cj0183* promoter motifs was experimentally investigated by generating substitutions and deletions in these regions (Fig. 2), and performing subsequent transcriptional fusions of the mutated promoters with a promoterless *lacZ* reporter gene in pMW10 [17]. Effects of mutations made in the promoter region were then quantitatively assessed based on the activity of the β-galactosidase enzyme produced.

The *C. jejuni* 81-176 *cj0183* promoter contains an optimal -10 region (TATAAT) compared to the consensus *E. coli* and *C. jejuni* sequence (TATAAT). Substitution within this motif by an unrelated sequence GGATCC rich in GC (Subs/restr -10) abolished β-galactosidase activity completely (Fig. 3). The study by Petersen et al. [10] indicated that the -10 motif is a highly conserved; thus, it is not surprising that substitution within this region seriously influences the activity of the promoter.

The predicted -35 motif (TTCAAATnTT) does not match the *E. coli* consensus sequence, but is similar to the predicted canonical *C. jejuni* -35 motif TTTAAGTnTT [17]. Alteration of the *cj0183* -35 motif to the consensus sequence in the Subs/cons -35 strain increases β-galactosidase activity approximately twofold (Fig. 3). In contrast, as mentioned above, deletion of the -35 region decreases threefold β-galactosidase activity. Our studies determined that the -35 region is not essential for promoter activity; its removal significantly reduces, but does not abolish the activity of the examined promoter sequence. However, the promoter activity increased significantly above 1,000 Miller units, when the -35 region was constituted by the consensus sequence proposed by Wösten et al. [17]. Data published by Petersen et al. [10] describing an in silico analysis of the predicted promoter sequences of *C. jejuni* and *H. pylori* show a weakly conserved -35 motif. Despite that in *C. jejuni* the -35 region is not highly conserved, its strong compatibility with the consensus sequence increases promoter strength.

The spacer between -35 (TTCAAATnTT) and -10 (TATAAT) motifs in the *cj0183* promoter counts 19 bp. Within this region we have found a -16 motif. The *C. jejuni* 81-176 *cj0183* promoter contains a suboptimal -16 region (TTAATTGT) compared to the consensus *C. jejuni* sequence (TTTTTTTG). Alteration of the *cj0183* -16 motif to the consensus sequence in the Subs/cons -16 mutant does not significantly affect β-galactosidase activity (Fig. 3). Our results allowed to establish that the -16 motif is important for transcription of the *cj0183* gene. Mutation of the -16 motif to a AGATCTT sequence in Subs/restr -16 abolished β-galactosidase activity completely (Fig. 3).

The importance of the -16 TGn (TTTTTT TG) motifs in transcription initiation has been well documented in other bacteria [1, 4, 14]. The extended -10 TGn motif seems to play an important role in *C. jejuni* transcription since a third of predicted by Wösten et al. and about 13 % of computationally discovered (RSAT) *C. jejuni* promoters have this motif (Tables 3, 4). The TGn motif is found to compensate poor -10 hexamer and -35 element in *E. coli* [5, 9]. Detailed analysis of predicted *C. jejuni* promoters indicates a weaker -35 motif in promoters with TGn comparison with non-TG promoters. Furthermore, the -10 region resembles more the consensus of TG promoters (Table 4). This confirms the observation of Petersen et al. [10] that *C. jejuni* promoters contain a strongly conserved -10 region, but do not possess a conserved -35 region. Moreover, *C. jejuni* promoters have a strong periodic signal upstream of the -10 region that has not been reported before in bacterial promoters. It contains thymine bases every 10–11 bp and may play a role in environmental regulation of the gene expression level.

**Table 4:** Occurrence of dinucleotides nearby the extended -10 motif and -35 motif of *C. jejuni* promoters

	-35 motif									TG motif			-10 motif					
	T	n	A	A	G	T	n	T	T	T	G	n	T	A	T	A	A	T
All promoters																		
Promoters identified by Wösten [17]	66 %		57 %		33 %			38 %		33 %			85 %		33 %		47 %	
Promoters identified in this study	94 %		93 %		54 %			16 %		13 %			92 %		86 %		89 %	
TG promoters																		
Promoters identified by Wösten [17]	57 %		42 %		28 %			42 %		100 %			85 %		42 %		57 %	
Promoters identified in this study	84 %		84 %		42 %			21 %		100 %			100 %		89 %		100 %	
Non-TG promoters																		
Promoters identified by Wösten [17]	71 %		64 %		35 %			35 %		0 %			85 %		28 %		42 %	
Promoters identified in this study	96 %		95 %		56 %			17 %		0 %			91 %		85 %		87 %	

The -16 region of the *cj0183* gene has two TG motifs—TAATTG TGTATAAT immediately upstream of the -10 hexamer at -13/-14 positions and TAAT TGTGTATAAT with a 2 nucleotide spacer (TGnn) at -15/-16 positions. The promoter in the Subs/cons -16 mutant (consensus -16 motif sequence) has a single nucleotide spacer between the -16 and -10 region (TGn) at -14/-15 positions and the same activity as the wild-type *cj0183* gene promoter (Figs. 2, 3). The *cj0183* promoter contains a G at the -13 position. Djordjevic [5] suggested significant conservation of this nucleotide in *E. coli*, but its importance has not been reported.

Further study may provide insight into the process of gene transcription in *C. jejuni* and may be relevant in understanding the molecular responses required to survive in the host. Therefore, to confirm the exact role of the -16 region and TG motif in *C. jejuni* gene transcription, single-base pair mutations in these regions will be required.

## References

[1] Burr T, Mitchel J, Kolb A, Minchin S, Busby S (2000). DNA sequence elements located immediately upstream of the -10 hexamer in *Escherichia coli* promoters: a systematic study. Nucleic Acids Res.

[2] Carvalho E, Barbosa AS, Gómez RM, Cianciarullo AM, Hauk P, Abreu PA, Fiorini LC, Oliveira ML, Romero EC, Gonçales AP, Morais ZM, Vasconcellos SA, Ho PL (2009). Leptospiral TlyC is an extracellular matrix-binding protein and does not present hemolysin activity. FEBS Lett.

[3] Chen S, Bagdasarian M, Kaufman MG, Bates AK, Walker ED (2007). Mutational analysis of the *ompA* promoter from *Flavobacterium johnsoniae*. J Bacteriol.

[4] Davies BJ, de Vries N, Rijpkema SG, van Vliet AHM, Penn CW (2002). Transcriptional and mutational analysis of the *Helicobacter pylori* urease promoter. FEMS Microbiol Lett.

[5] Djordjevic M (2011). Redefining *Escherichia coli* σ^70^ promoter elements: -15 motif as a complement of the -10 motif. J Bacteriol.

[6] Jeon B, Itoh K, Ryu S (2005). Promoter analysis of cytolethal distending toxin genes (cdtA, B, and C) and effect of a luxS mutation on CDT production in *Campylobacter jejuni*. Microbiol Immunol.

[7] Kim M, Hwang S, Ryu S, Jeon B (2011). Regulation of *perR* expression by iron and PerR in *Campylobacter jejuni*. J Bacteriol.

[8] Korlath JA, Osterholm MT, Judy LA (1985). A point-source outbreak of campylobacteriosis associated with consumption of raw milk. J Infect Dis.

[9] Mitchell JE, Zheng D, Busby SJW, Minchin SD (2003). Identification and analysis of ‘extended -10’ promoters in *Escherichia coli*. Nucleic Acid Res.

[10] Petersen L, Larsen TS, Ussery DW, On SL, Krogh A (2003). RpoD promoters in *Campylobacter jejuni* exhibit a strong periodic signal instead of a -35 box. J Mol Biol.

[11] Sałamaszyńska-Guz A, Klimuszko D (2008). Functional analysis of the *Campylobacter jejuni cj0183* and *cj0588* genes. Curr Microbiol.

[12] Thies FL, Weishaupt A, Karch H, Hartung HP, Giegerich G (1999). Cloning, sequencing and molecular analysis of the *Campylobacter jejuni* groESL bicistronic operon. Microbiology.

[13] van Helden J (2003). Regulatory sequence analysis tools. Nucleic Acids Res.

[14] Voskuil MI, Chambliss GH (1998). The -16 region of *Bacillus subtilis* and other gram-positive bacterial promoters. Nucleic Acids Res.

[15] Wassenaar TM, Fry BN, van der Zeijst BA (1993). Genetic manipulation of *Campylobacter*: evaluation of natural transformation and electro-transformation. Gene.

[16] Wooldridge KG, Jones M, Morris S, Carrasco M, Powel H, Forsythe S, Ala’Aldeen D (2004) Two-toxins related genes of *C. jejuni* have a role in colonisation of the chickens. CHRO: 78

[17] Wösten MM, Boeve M, Koot MG, van Nuenen AC, van der Zeijst BA (1998). Identification of *Campylobacter jejuni* promoter sequences. J Bacteriol.

